# Hypercholesterolemia and 3-Hydroxy 3-Methylglutaryl Coenzyme A Reductase Regulation during Ageing

**DOI:** 10.1100/tsw.2009.81

**Published:** 2009-07-04

**Authors:** Laura Trapani, Valentina Pallottini

**Affiliations:** Department of Biology, University of Roma Tre, Roma, Italy

**Keywords:** aging, hypercholesterolemia, HMG CoA reductase

## Abstract

We present here a brief description of the path that cholesterol covers from its intestinal absorption to its effect exerted on some enzyme regulation. Some mechanisms underlying hypercholesterolemia onset and, in particular, the role and the regulation of 3-hydroxy 3-methylglutaryl Coenzyme A reductase (HMGR) during adult life and during aging, have been described. In addition some pharmacological interventions to control proper HMGR regulation and, in turn, cholesterol homeostasis maintenance will be introduced.

## INTRODUCTION

Cholesterol is an essential component of the membranes of most cells in the body. It is extremely important that all cells have a continuous supply of this important molecule for the synthesis of new membranes, for the turnover of lipids in existing membranes, and for the biosynthesis of certain products such as steroid hormones and bile acids. To meet this need, a complex series of biosynthetic mechanisms and transport processes have evolved to maintain cholesterol homeostasis across the body as a whole and across each of the major organ systems. An elevated cholesterol amount within cells, however, may lead to pathological consequences[1]. This is particularly true for cells of the artery wall, where accumulation of cholesterol initiates atherosclerotic cardiovascular diseases[2]. Therefore, the body relies on a complex homeostatic network to modulate the availability of cholesterol for tissues. This network operates on both the cellular level and within the plasma compartment. Cholesterol is both synthesized by cells and taken in with food. The liver is the principal site for cholesterol homeostasis maintenance[3], carried out in many mechanisms, such as biosynthesis, via 3-hydroxy-3-methylglutaryl coenzyme A reductase (HMGR, E.C. 1.1.1.34) activity, uptake through low-density lipoprotein receptors (LDLr), lipoprotein release in the blood, storage by esterification, degradation, and conversion into bile acids[4]. Since HMGR is a central enzyme in cholesterol homeostasis, it is the target for pharmacological treatment of hypercholesterolemia. HMGR is differently regulated during all stages of life and during aging, in particular, this enzyme results to be completely deregulated.

Cholesterol homeostatic regulation, the age-dependent disruption of cholesterol homeostasis, and HMGR modulation both in adult life and during aging will be described in the following.

## CHOLESTEROL ABSORPTION, SYNTHESIS, AND TRANSPORT

One of the sources for the acquisition of cholesterol by animals and man is the absorption of sterol ingested in the diet. During digestion, cholesterol esters are broken into unesterified cholesterol and long-chain fatty acids; such monomers of unesterified cholesterol can diffuse directly up to the microvillus border of the intestinal epithelial cell and be absorbed passively[5].

However, absorption by this mechanism is extremely slow because of the thick diffusion barrier that exists between the bulk solution of the intestinal content and the microvillus border of the absorptive cell. Hence, the cholesterol molecules become incorporated into bile acid micelles, which act as a shuttle to deliver large amounts of sterol to the region of the aqueous-microvillus interface from which absorption takes place. Hence, rapid cholesterol absorption is critically dependent on the presence of adequate concentrations of bile acids within the intestinal lumen[6,7]. After entering the enterocytes, approximately half of the cholesterol molecules move to the endoplasmic reticulum (ER) where cholesterol is esterified by acyl-CoA:cholesterol acyltransferase (ACAT) before incorporation into nascent chylomicron (CM) particles produced by the small intestine soon after a meal[8,9]. CMs carry a lot of triglycerides (TGs) as well. These particles are released from the base of the intestinal epithelial cell and enter the intestinal lymphatic vessels, where they interact with high-density lipoprotein (HDL) to acquire apoproteins C and E[10]. Although the liver is the most important site for such whole-body synthesis, all organs in the body are capable of significant rates of cholesterol synthesis[5].

The 27-carbon tetracyclic cholesterol molecule is synthesized from acetate in a series of ~30 enzymatic reactions. The rate-limiting enzyme of the pathway is the HMGR, an integral membrane protein spanning eight times the ER and able to reduce 3-hydroxy-3-methylglutaryl Coenzyme A (HMG-CoA) to mevalonate. This enzyme harbors a conserved sterol-sensing domain (SSD) with five membrane-spanning helices that is shared by other proteins implicated in sterol regulation[11]. The SSD of HMGR is critical both for the association of the protein with another ER resident protein, Insulin Induced Gene (Insig), and for its degradative regulation[12]. The production of the first sterol in the cascade, lanosterol, is catalyzed by squalene cyclase. The subsequent 20 steps constitute the postlanosterol part of cholesterol biosynthesis in which double bonds are reduced, their positions altered, and methyl groups removed. Importantly, isoprenoid intermediates in the presqualene half of the pathway serve as precursors not only for cholesterol, but also for a number of other biomolecules involved in transcription (isopentenyl tRNAs), protein *N*-glycosylation (dolichol), protein prenylation (farnesyl and geranylgeranyl moieties), and mitochondrial electron transport (ubiquinone), all indispensable molecules for cellular physiological functions (Fig. 1)[13]. The first seven enzymes of cholesterol biosynthesis are soluble proteins apart from HMGR, which is, as described above, an integral ER membrane protein. HMGR and some of the other prelanosterol biosynthesis enzymes are also present in peroxisomes[14], but the enzyme directly following HMGR, mevalonate kinase, is cytosolic[15]. The postlanosterol enzymes are localized to the ER or its extensions, nuclear envelope, or lipid droplets. The significance of this complex subcompartmentalization of the cholesterol biosynthetic pathway remains poorly understood[13].

With both cholesterol absorption and cholesterol synthesis taking place, it is apparent that it is necessary to move cholesterol between the different organs in order to maintain internal cholesterol balance under circumstances of changing cholesterol needs. Such cholesterol movement is mediated by different lipoproteins that target the cholesterol to specific organs within the body. For example, most of the cholesterol absorbed from the diet by the intestine is incorporated, as described above, into CMs that, ultimately, are taken up by the liver. The rate of cholesterol synthesis in the liver varies inversely with the amount of dietary cholesterol reaching it in the CMs[16]. Hence, sterol synthesis within the intestinal-hepatic axis tends to accommodate to changing rates of dietary cholesterol entrance into the body. Cholesterol moves out of the liver largely carried in VLDL (very low-density lipoproteins) that are gradually converted into IDL (intermediate-density lipoproteins) and LDL (low-density lipoproteins). Cells that have a demand for cholesterol bind LDL through their LDL receptors (LDLr) and then take up the complete particle through receptor-mediated endocytosis. This type of transport is mediated by depressions

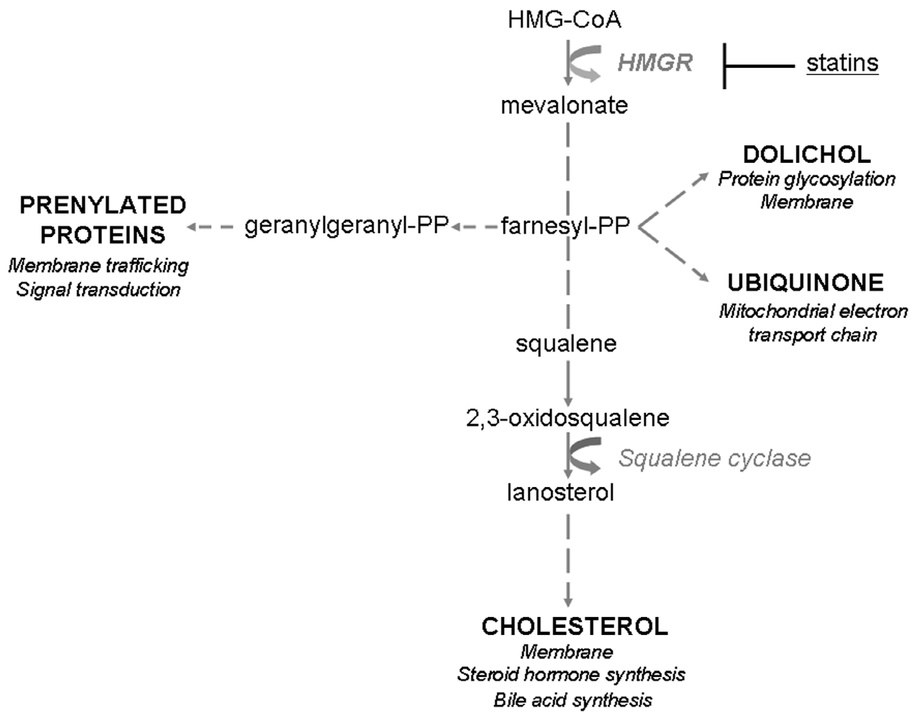

**FIGURE 1.** Schematic illustration of the biosynthetic pathway of HMGR end products.

in the membrane ("coated pits"), the interior of which is lined with the protein clathrin. After LDL binding, clathrin promotes invagination of the pits and pinching off of vesicles ("coated vesicles"). The clathrin then dissociates off and is reused[17]. After fusion of the vesicle with lysosomes, the LDL particles are broken down, and cholesterol and other lipids are used by the cells. The HDLs also originate in the liver. They return the excess cholesterol formed in the tissues to the liver. While it is being transported, cholesterol is acylated by lecithin cholesterol acyltransferase (LCAT). The cholesterol esters formed are no longer amphipathic and can be transported in the core of the lipoproteins. In addition, HDLs promote CM and VLDL turnover by exchanging lipids and apoproteins with them[18].

## HYPERCHOLESTEROLEMIA AND AGING

It is widely accepted that abnormal levels of lipids and/or lipoproteins in blood are a modifiable risk factor for coronary artery disease (CAD)[19,20].

The term CAD refers to pathologic changes within the coronary artery walls that diminish blood flow through these vessels. CAD can cause myocardial ischemia and possibly lead to acute myocardial infarction by three mechanisms: profound vascular spasm of the coronary arteries, formation of atherosclerotic plaques, and thromboembolism. In particular, the atherosclerotic plaque consists of a lipid-rich core covered by an abnormal overgrowth of smooth muscle cells topped off by a collagen-rich connective tissue cap. As the plaque forms, it bulges into the vessel lumen. Typically, the initial stage of atherosclerosis is characterized by the accumulation beneath the endothelium of excessive amounts of LDL in combination with a protein carrier. As LDL accumulates within the vessel wall, LDL becomes oxidized, primarily by oxidative wastes produced by the blood vessel cells. In response to the presence of oxidized LDL, the endothelial cells produce chemical-attracting monocytes that trigger a local inflammatory response. Once the monocytes leave the blood and enter the vessel wall, they settle down and become macrophages that phagocytize the oxidized LDL; thus, they appear foamy and accumulate beneath the vessel lining, forming the earliest type of an atherosclerotic plaque. The disease progresses as smooth muscle cells within the blood vessel wall migrate from the muscular layer of the blood vessel to a position on top of the lipid accumulation, just beneath the endothelium. At their new location, the smooth muscle cells continue to divide and enlarge, producing atheromas. Together with lipid-rich core and overlying smooth muscle, they form a maturing plaque. As it continues to develop, the plaque progressively bulges into the lumen of the vessels; thus, the blood flow and, in turn, the oxygen availability for the myocardial cells downstream of the obstructed vessels highly decrease, leading to heart ischemic events[21].

The importance of lipid levels in older adults is controversial. Several studies have suggested that the association between cholesterol concentrations and atherosclerotic CAD weakens with age, and that there is little potential benefit from screening and treating older adults for dyslipidemia[22,23]. In contrast, other reports suggest that lipoprotein levels remain a significant risk factor for CAD in older people and that the treatment of dyslipidemia in older people may have a greater impact on CAD mortality than in younger people because the total attributable risk from dyslipidemia is greater in the older age group[24,25]. One reason for the controversy about screening and treating hyperlipidemia in older adults is the relatively small number of participants (especially among the very old), and the lack of details about the demographic characteristics and about health status of the sample[19]. Furthermore, few studies have examined the power of lipids as risk factors for cardiovascular disease in older adults.

Among people aged 65 years and older with prior myocardial infarction in the Framingham Study, serum total cholesterol was most strongly related to death from CAD and to all-cause mortality[26]. An increase of 10 mg/dl of serum total cholesterol significantly increased the relative risk of new coronary events by 1.12 times in men and by 1.12 times in women[27]. In 1,793 older men and women, mean age 81 years, there was a 1.28 times significantly greater probability of having CAD for an increment of 10 mg/dl of serum LDL cholesterol[28]. A low serum HDL cholesterol is also a risk factor for new coronary events in older men and women[29]. In the Framingham Study, a low serum HDL cholesterol was a more powerful predictor of new coronary events than was serum total cholesterol; a decrease of 10 mg/dl of serum HDL cholesterol significantly increased the relative risk of new coronary events by 1.7 times in men and by 1.95 times in women. In older men and women, there was a 2.56 times significantly greater probability of having CAD for a decrease of 10 mg/dl of serum HDL cholesterol[28]. Hypertriglyceridemia has been shown to be a risk factor for new coronary events in older women, but not in older men[27,29]. The presence of serum triglycerides was not an independent risk factor for new coronary events in older men and was a very weak independent risk factor for new coronary events in older women.

The mechanisms behind this age-related dyslipidemia are still incompletely characterized. It has to be taken into account that aging is characterized by the perturbance of the homeostasis or, in other words, by the drifting away from the properly differentiated state[30] that leads to changes in biochemical composition in tissues (the percentage of adipose tissue increases with age)[31,32,33], to a reduced ability to respond adaptively to environmental stimuli[34], and to an increased susceptibility to disease[35]. Of particular interest is the finding of a gradual decline in the fractional clearance of LDL from the circulation with age[36,37,38] and evidence of the reduced expression of hepatic LDLr with increasing age in some species[39,40,41].

The capacity for body cholesterol removal through the conversion of cholesterol to bile acids is also progressively reduced with age and a decrease in the activity of the rate-limiting enzyme in bile acid biosynthesis, cholesterol 7α-hydroxylase (C7αOH), has been demonstrated in the aging rat[42]. In addition, there is some evidence that the synthesis of apolipoprotein B-100 in VLDL may be increased with age[43].

An interesting hypothesis states that the critical changes in cholesterol and lipoprotein metabolism depend on the progressive decrease in growth hormone (GH) secretion, which occurs with normal aging[44,45,46]. It has been demonstrated in hypophysectomized rats that GH has an important role in cholesterol homeostasis[44], both by modulating the expression of hepatic LDLr[47,48,49,50] and by controlling the activity of C7αOH[51]. In addition, it has been demonstrated that GH replacement therapy improves lipid profile by increasing the removal of VLDL apoB. Although GH therapy stimulates VLDL apoB secretion, this is offset by the increase in the VLDL apoB clearance rate, which is probably due to its effects in up-regulating LDLr and modifying VLDL composition[52]. The increase of plasma cholesterol due to the reduced elimination of cholesterol as bile acids and the decreased receptor-mediated clearance of plasma LDL can be reversed by treatment with GH.

The level of intestinal absorption of cholesterol may also contribute to the development of hypercholesterolemia[1].

Given that several epidemiologic studies have identified the elderly population as having a high risk for cardiovascular events, risk-factor modification plays an important role in an attempt to reduce adverse cardiovascular events. On the other hand, it has to be taken into consideration that low cholesterol has also been associated with risk of cancer, findings that lead to concern that population interventions to lower cholesterol could lead to an increased risk of cancer, but careful investigation revealed that the low cholesterol was a result of the preclinical presentation of cancer[53].

Several drugs have been developed as cholesterol-lowering therapeutic agents. Some of them, such as statins, show unpleasant side effects. Thus, research turns the attention on other hypocholesterolemic agents and a growing plethora of compounds are appearing in the scientific literature. Actually, most of them reduce serum cholesterol affecting intestinal cholesterol absorption or by mechanisms not completely defined that produce effects reducing cardiovascular risk factors[54,55,56,57,58,59,60].

## HMGR REGULATION IN ADULT LIFE AND IN AGING

In order to comprehend the probable causes that trigger hypercholesterolemia, the mechanisms underlying the regulation of HMGR, the key and rate-limiting enzyme of cholesterol biosynthesis, will be described in detail.

As mentioned before, HMGR is the central enzyme of the cholesterol biosynthetic pathway; thus, it is tightly regulated[61].

Encoded by the *hmgr* gene located on chromosome 5, HMGR consists of a single polypeptide chain of 888 amino acids. The aminoterminal 339 residues are membrane bound and reside in the ER, whereas the catalytic activity of the protein resides in its cytoplasmic, soluble C-terminal portion (residues 460–888). A linker region (residues 340–459) connects the two portions of the protein[62].

The catalytic portion of HMGR forms a tetramer whose individual monomers wind around each other in an intricate fashion. In the tetramer, the monomers are arranged in two dimers, each of which has two active sites. Each monomer consists of three domains: a helical N-terminal domain, a large “L-domain” (the HMG-CoA binding site), and a small “S-domain” (the NADP[H] binding site)[62].

The enzyme is subjected to both short- and long-term regulations.

Short-term regulation of HMGR is achieved principally by phosphorylation and dephosphorylation reactions, both able to affect enzyme activity. The phosphorylation of the enzyme’s residue S872 decreases HMGR catalytic activity. Since S872 is located close to the catalytically important residue H866 in the primary structure, it was proposed that the phosphoserine interacts directly with H866 and abstracts its imidazolium proton[63]. This suggests that phosphorylation is likely to result in a decrease in affinity for NADPH. Removal of the phosphate by HMGR phosphorylase reactivates the enzyme[64,65]. *In vitro*, HMGR may be phosphorylated by several protein kinases: AMP-activated protein kinase (AMPK)[66], protein kinase C[64], and a calmodulin-dependent protein kinase[67]. The AMPK appears to be the major HMGR kinase in the liver, where cholesterologenesis takes place. AMPK is a heterotrimeric serine/threonine kinase consisting of a catalytic α subunit and regulatory β and γ subunits[68]. AMPK is activated by phosphorylation of the subunit α at a specific threonine residue (Thr172)[69]. AMPK is also able to phosphorylate and inactivate the acetyl-CoA carboxylase, the rate limiting enzyme of fatty acid biosynthesis[70].

HMGR activation (dephosphorylation) is operated by protein phosphatase 2A (PP2A), an abundant cellular serine/threonine phosphatase that regulates a significant network of cellular events. The PP2A

holoenzyme is a heterotrimer containing a 65-kDa regulatory A subunit, a 36-kDa catalytic C subunit, and various kinds of B subunit[71].

Beside short-term regulation, HMGR is subject to transcriptional, translational, and post-translational control[72]. It can result in changes of over 200-fold in enzyme levels as a function of intracellular sterol amount and in dependence on cholesterol uptake by LDLr[61].

To monitor levels of membrane sterols, cells employ, beside HMGR, another membrane-embedded protein of the ER, Scap (sterol cleavage activating protein), which shares the polytopic intramembrane sequence SSD. Scap is an escort protein for sterol regulatory element binding proteins (SREBPs), membrane-bound transcription factors able to induce the expression of genes required for the synthesis and the uptake of cholesterol, such as, among others, HMGR and LDLr[73,74]. In sterol-deprived cells, Scap binds SREBPs and escorts them from the ER to the Golgi apparatus where the SREBPs are proteolytically processed to yield active fragments that enter the nucleus and induce their target genes' expression[75]. When cholesterol builds up in ER membranes, the Scap/SREBP complex fails to exit the ER, proteolytic processing of SREBPs is abolished, and transcription of target genes declines[76]. ER retention of Scap/SREBP is mediated by sterol-dependent binding of Scap/SREBP to the above-mentioned Insig protein[61]. The intracellular accumulation of sterols induces HMGR to bind Insig, which promotes the enzyme ubiquitination and proteasomal degradation[12]. Mammalian genomes contain two *Insig* genes that encode Insig-1 and Insig-2, which display a similar tissue expression pattern. Insigs contain six transmembrane segments. Insig-1, but not Insig-2, is a SREBP target gene and has a short half-life due to its degradation by the proteasome. However, to date, these ER resident proteins are functionally interchangeable[77]. The reciprocal regulation of these two proteins is still unknown.

Several hormones act to alter the expression of hepatic HMGR in animals. These include insulin, glucagon, glucocorticoids, thyroid hormone, and estrogen. Insulin stimulates HMGR activity likely by increasing the rate of transcription, whereas glucagon acts by opposing this effect. Hepatic HMGR activity undergoes a significant diurnal variation due to changes in the level of immunoreactive protein primarily mediated by changes in insulin and glucagon levels. Thyroid hormone increases hepatic HMGR levels by acting to increase both transcription and stability of the mRNA. Glucocorticoids act to decrease hepatic HMGR expression by destabilizing reductase mRNA[78]. Data about estrogen action on HMGR are still debated; in fact, some authors assess that estrogens act to increase hepatic HMGR activity primarily by stabilizing the mRNA and that deficiencies in those hormones that act to increase hepatic *hmgr* gene expression lead to elevations in serum cholesterol levels[78]. Other data, obtained in the DLD1 cell line, show that estrogens induce an early increase of LDLr, at both mRNA and protein level, and later decrease HMGR activity and protein expression[79]. More recent data suggest that sex differences in HMGR expression and regulation depend on variation in regulatory proteins and are related to estrogen presence. The authors sustain that HMGR levels and activity are lower in adult females than in males. This feature seems to depend on estrogen-dependent Insig-2 levels that are able to regulate both the transcription and degradation of the enzyme[80].

Owing to the enormous consequences associated with aging, significant efforts have been invested to get a better picture of this important life process. Current research directed toward a fundamental understanding of biological aging mechanisms has provided valuable insights into the molecular basis of age-related deterioration. One of the critical problems associated with biological aging is the involvement of age-related diseases, such as CAD. Hypercholesterolemia is a risk factor for CAD; thus, the study of the mechanisms underlying the increased cholesterol content during aging is essential to discover specific intervention points. During aging, hepatic lipid modifications occur[1].

Few studies are present in the literature on the HMGR physiological regulation during this specific stage of life. In particular, experiments conducted on aged rats showed increased plasma cholesterol levels and hepatic cholesterol synthesis accompanied by a full activation of HMGR[81,82]. In fact, HMGR results to be completely dephosphorylated and activated[81,82,83].

Although controversial, it has been reported that during aging, the mitochondria produce significantly higher levels of superoxide ions and that the ability of cells to remove such a deleterious surplus of free radicals is strongly reduced[84,85]. The age-related total activation of the HMGR has been associated with a rise in reactive oxygen species (ROS)[86,87]. The proposed model is that high ROS levels should induce both p38 and AMPKα activation. In turn, p38 could determine an increase of PP2A association with the HMGR, which leads to dephosphorylation and full activation of that enzyme. The AMPKα phosphorylating activity could be impaired by the enhanced association of PP2A with the reductase (Fig. 2). Moreover, the $H_2O_2$-stimulated HepG2 cell line shows that the ROS effect on the HMGR dephosphorylation is mediated by the activation of the p38/MAPK pathway[83].

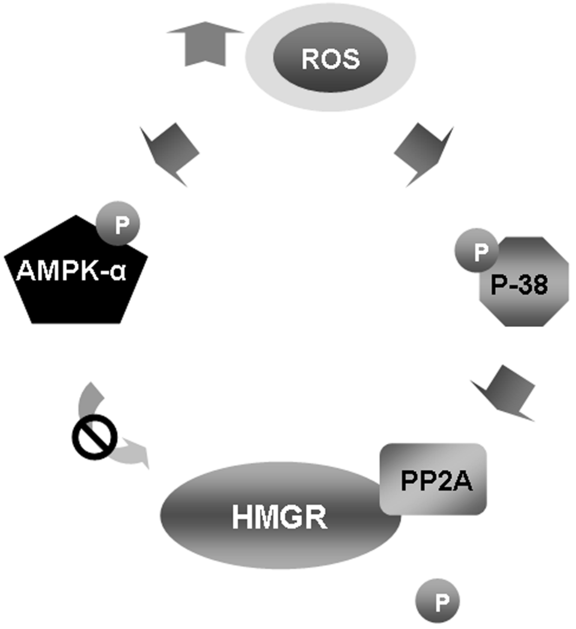

**FIGURE 2.** Proposed model of ROS-induced HMGR full activation state. The figure shows the proposed mechanism operated by ROS in inducing hepatic HMGR dephosphorylation. ROS increase is able to activate both AMPKα and p38, which in turn triggers the PP2A phosphorylating activity on HMGR, thereby activating it. PP2A action thus impairs AMPKα phosphorylating action on HMGR.

Such age-related modifications are prevented by caloric restriction (CR)[81,82], which is a dietary manipulation consisting of a decreased food intake[88]. CR extends the life span by slowing and/or delaying the aging processes; however, the underlying biological mechanism responsible for this life extension is still not completely known, although many hypotheses have been proposed[88].

Besides HMGR phosphorylation/dephosphorylation processes, HMGR long-term regulation turns out to be affected by aging as well.

Age-related hormonal variation and sensitivity induce a decreased ability to maintain homeostatic potential, and are always associated with a modified content or functionality of some molecules. In particular, it has been underlined that the decreased insulin sensitivity occurring in aging can lead to changes in the levels of some factors involved in cholesterol metabolism, such as Insig-1 protein.

Age-related Insig reduction determines a decreased degradation rate of the HMGR[41,89]. All these modifications are paralleled by a lower LDLr exposition on cellular membrane[82]. Both LDLr low membrane exposition and HMGR full activation state concur to age-related hypercholesterolemia.

## CONCLUSION AND FUTURE PERSPECTIVES

Great advances have been made in the comprehension of molecular control of sterol synthesis; above all, HMGR activity regulation has been an attractive target for intervention in the pharmacological treatment of hypercholesterolemia. A decrease in cholesterol synthesis in cells leads to a homoeostatic response, involving up-regulation of cell-surface receptors that bind atherogenic lipoproteins such as LDL and VLDL. Bound lipoproteins are taken up into cells and degraded[41]. This reduction in circulating atherogenic lipoproteins helps to explain the clinical utility of HMGR inhibitors (statins).

HMGR inhibition determines, as well as cellular and plasmatic cholesterol level reduction, the decrease of products sharing with cholesterol the same biosynthetic pathway (i.e., ubiquinone, prenylated proteins, dolichol).

This event can produce alterations of cell physiology. In particular during aging, this picture could make the precarious cell homeostasis worse. Thus, the restoration of the proper HMGR activation state, without completely blocking the enzyme, would determine physiological synthesis of cholesterol within cells and, in turn, the correct LDLr amount on membrane in dependence on cell sterol content, as described by the classical model generally accepted[77]. It has been demonstrated that CR is able to prevent age-related hypercholesterolemia by regulating HMGR activation state and LDLr membrane exposition, but a CR dietetic habit is very hard to adapt to human beings; thus, the identification of a valid alternative has to be taken into account. For example, it has been demonstrated that a diet supplemented with Omega 3 fish oil can represent a valid alternative to CR regarding the maintenance of cholesterol homeostasis[60]. Moreover, the identification of Scap and Insig as sterol-binding proteins in mammalian cells added new molecular details to SREBP pathway regulation and, in turn, to HMGR levels. A lot of work has to be done in order to comprehend the relationship between hormonal modifications, their effect on transcription factors, and cholesterol metabolism in different physiological and pathological conditions.

During aging, Insig proteins decline; thus, more detailed studies will be required both to define the specific roles of each Insig proteins and to determine the metabolic consequences of their reciprocal regulation. Since Insigs are required for feedback regulation of SREBP proteolytic processing and HMGR degradation, these proteins could represent a new target for pharmacological intervention in order to maintain blood cholesterol levels in the optimal range, thus reducing CAD as the main risk factor.

## ACKNOWLEDGMENT

The authors wish to thank past and present members of their laboratories who contributed, with data and discussions, to the ideas presented here. In particular, the invaluable and dedicated work of our mentor Prof. Anna Trentalance is warmly acknowledged.
